# Book Reviews

**Published:** 1804-07-01

**Authors:** 


### CRITICAL ANALYSIS

### OF THE

### RECENT PUBLICATIONS

### ON THE

### DIFFERENT BRANCHES OF PHYSIC, SURGERY, AND MEDICAL PHILOSOPHY.

---

*Observations on the Treatment of Scirrhous Tumours and Cancers of the Breast.* By JAMES NOOTH, Surgeon Extraordinary to His Royal Highness the Duke of Kent, &c. &c. Octavo, pp. 101. Bath, 1804.

AFTER an historical introduction, the author commences his observations with an anatomical description of the breast, and particularly those parts in which cancer originates; he then explains the appearances and symptoms of the incipient disease, and its usual progress. The experiments he tried on himself by inoculation with diseased fluids, are a sufficient proof of the intrepidity of his investigations. He next proceeds to mention the different kinds of treatment which have excited the hopes of practitioners for a time, but which have been found ineffectual on more extensive experience. Among these, *cicuta*, *mercury*, *antimonials*, *flores martiales*, *belladonna*, *solanum*, *arsenic*, and *electricity* are mentioned, all of which, particularly the last, have been useful in tumours of the breast, though they will not cure *cancer*.

Mr. N. then examines the question, whether scirrhous affections proceed from any predisposing state of the constitution or from accidental causes, and determines in favour of the latter opinion, and on this determination he properly founds the importance of extirpation. A minute account of the operation follows, with the subsequent treatment and observations on the *prognosis;* to which are subjoined, a number of cases to confirm their utility and propriety.

The author concludes his truly practical pamphlet with the means of alleviating those cases of open cancer which cannot be relieved by the foregoing methods.

"Strumous swellings of the breast, and small encysted tumours, are easily distinguished by surgeons. Indeed, it seems barely possible to mistake those affections for schirrous tumours, all attendant symptoms being duly considered.

"The advanced stages of cancer, where ulceration has taken place, become extremely painful to the miserable patient, and resist the most rational treatment which can be suggested by medical skill or surgical assistance. A proper attention to such diet as will not stimulate must be paid, and the bowels should be kept, by gentle gentle means, moderately lax; opiates in small doses, and frequently given, will procure *temporary* relief. No dressings, I think, yet employed by our best surgeons, have been found to be of essential service; but such as do not stimulate, yet exclude the atmospheric air, seem best calculated to avoid irritability; and it is always necessary to keep the sores as clean as possible.

"No attempt to restrain fungous excrescences can be made without exciting, *in some degree*, irritability; therefore such as are most gentle in their effects should be preferred. These circumstances are more obstinate and distressing than any others with which a surgeon ever has to contend. He might, *in a slight degree*, palliate distress, but cannot remove it.

"I shall now," says Mr. N. "with much satisfaction, offer the result of some experiments with the carbonic acid gas on cancerous ulcers of the breast. I am aware that this gas has been employed for the same purposes by many gentlemen of the most respectable abilities; but, I trust, the mode of application here described, will be found more effectually convenient than any other hitherto adopted.

"A poor woman was my patient at the Infirmary, and a general consultation was held on her case; when we were unanimously of opinion, that no relief could be obtained by extirpation. I therefore proposed the application of the carbonic acid gas to the ulcer, as it might probably afford some mitigation of her sufferings; which idea was warmly supported by Dr. Ewart, and readily acceded to by the other medical gentlemen.

"On the following day, Mr. White, apothecary to the Infirmary, happily conceived a plan for keeping it always in application with the sore, by means of a small bladder, which was to be affixed to the breast by *sticking plaster*. The neck of the bladder was cut off, so as to make a circular aperture into it, of such dimensions as to correspond nearly with the size of the ulcer of the breast. A round hole of the same size was cut in a piece of soft leather, spread with adhesive plaster, and large enough to surround the ulcer. The cut end of the bladder was introduced through the hole in the leather, and its edges folded back and stuck to the plaster on the opposite side, forming somewhat the shape of a round hat, the plaster resembling the rim, and the bladder, when distended, the crown. In order the more effectually to secure the attachment of the bladder to the plaster, and to make it air-tight, the end had cuts round it half an inch deep, and as much from each other to make it sit smoothly; and narrow slips of plaster were applied round their junction, both within and without. The large plaster was then fixed on the mamma, the aperture in its centre, with the bladder fixed to it, being placed exactly over the ulcer, no part of which was touched by the plaster. A small orifice was made at the fundus of the bladder, sufficient to admit a tube of about a quarter of an inch in diameter, which communicated with the top of an inverted cylinder, suspended upon water, which cylinder was filled with carbonic acid gas. The bladder being closely squeezed, to to expel the atmospheric air it contained, and the above mentioned tube being inserted into the orifice formed to receive it, and tied by a ligature passed over the bladder, the inverted cylinder was pressed down in the water, so that the carbonic acid gas was made to rush through the tube, and distend the bladder, which was tied above the tube, to prevent the escape of the gas. As soon as the bladder was collapsed, so as to shew that much of the gas had evaporated, it was filled in the same manner as before; and this operation was repeated twice or three times a day, as it appeared necessary.

"It is a proof of this simple apparatus fully answering its purpose, that the bladder, when filled at night, was found to contain a considerable quantity of the gas on the following morning. When the carbonic gas was thus applied to the sore, it first occasioned a sensation of coldness, which lasted for a few minutes, and was afterwards succeeded by a glowing warmth, which continued more than half an hour; the same sensations were uniformly expressed by the patient after each successive application of the gas."

---

*Medical Sketches of the Expedition to Egypt from India, by* JAMES M'GREGOR, A. M. *Member of the Royal College of Surgeons, Surgeon to the Royal Regiment of Horse Guards, and lately Superintending Surgeon to the Indian Army in Egypt.*

UNDER the title of Sketches we have the history of the diseases of the army which came from India to co-operate with the English Army in Egypt.

After remaining very healthy during a long period on shipboard, and after a very perilous navigation of the Red Sea, the Indian Army, consisting of about 8000 Europeans and Sepoys, landed at Rossier on the western shore of the Red Sea. In going from Rossier to Ghenné on the Nile in Upper Egypt, the Indian Army nearly followed the route travelled and described by Mr. Bruce.

From the want of water, from the intense heat, and from the hot winds, this march of an army, across the greater Desert, must have been attended with many difficulties. From Ghenné the army marched to Girge, the capital of Upper Egypt, was there embarked in boats, and after a navigation of nearly 400 miles, reached Cairo in August 1801.

Mr. M'Gregor, the Chief of the Medical Department of the Indian Army, informs us, "From the nature of the prevailing diseases, the campaign in Egypt was, in a particular degree, a service of danger. To their regret, the Indian Army arrived too late in Egypt, to share in any other dangers than those arising from the diseases of the country; and here the medical gentlemen had the post of honour. Intrepidity is more a military than a medical virtue; but seldom, I believe, has there been a greater display of it, than than among the medical officers in Egypt, whose duty it became to reside in the pest houses."

To shield him from the severity of criticism, Mr. M'Gregor offers this ingenuous apology for his authorship; it weighs with us, and we doubt not it will with readers in general, "The life of a medical man in the army is at no time very favourable to literary pursuits; mine has been peculiarly unfavourable; and I have had little time or opportunity, since I first entered the army, to attend to the ornaments of diction. For the last fifteen years of my life, mostly spent in the East-Indies, West-Indies, or at the Cape of Good Hope, sometimes at sea, sometimes on land, my time has been occupied in a laborious attention to my duty in the army."

Of three parts, into which this work is divided, the first gives the history of the expedition, the second treats of the causes of the diseases, and the third gives the history of diseases.

In assigning the causes of disease, Mr. M'Gregor expresses his confident hopes, that the modern chemistry will unfold many of them to us. Mr. M'G. we presume, was in India when he wrote his book; he seems equally sanguine on this subject, as many ingenious men in this country were some years ago.

The author adduces a particular instance of a fact which we are assured holds generally good, "That heat, though the degree of it be very considerably increased, unless combined with intemperance or some other cause, is very rarely the exciting cause of disease. At Rossier, and in crossing the desert, the degree of heat was very great, and both the officers and men, from Madras as well as Bengal, complained that it was more unsupportable than they had ever felt it in the hottest seasons. Yet, at the above period, the army enjoyed an uncommon degree of health, though they necessarily were much exposed to the sun: but their minds as well as their bodies were at this time exercised. Not only on crossing the desert, but for some time after we reached the Nile, at Ghenné, we all believed, that we were in the neighbourhood of a division of the French Army, and the Indian Army was for some time kept in the constant hopes of being engaged."

The chapter on the plague is the most valuable in the book, Mr. M'G's remarks are of the most consolatory nature; much, he thinks, may be done in the treatment of the disease, and every thing in the prevention, which in the Indian Army appears to have been attended with the most complete success. He remarks, "In Europe, but more particularly in Britain, much of the dread, and much of the real danger, that attended the most fatal diseases in this country are now done away, by the late improvements in medicine and in chemistry.

"The time was, that fever, when it broke out in different parts of England, proved little less fatal to whole villages and towns than the plague does in the countries where it resides. From the improved practice of the treatment of fever, but more from our knowledge of the means of destroying contagion and preventing its spreading spreading, the mortality from this class of diseases is now comparatively small. The small pox, that plague which once carried off so great a proportion of the population of Europe, now bids fair to be expunged from the catalogue of diseases. May we not indulge a hope, that, as the intercouse of civilized Europe with the countries of which the plague is now the scourge, becomes more regular and intimate, we may be enabled to extend to them our discoveries and improvements, and so direct them to the means of divesting the plague of its terrors, and reducing the mortality from it to the scale of that of fever and the small-pox in Europe." He brings testimony of the good effects of the nitrous fumigation. From the reports made to the author by the surgeons of the Indian Army, it appears that the treatment with mercury and nitrous acid was by far the most successful.

On the ophthalmia of Egypt, Mr. M'G. gives strong evidence of the disease being communicated by contagion, and he entertains the same opinion of Guinea-worm. This, to us, appears strange; but we recommend Mr. M'G's curious chapter on the subject. His experience in liver complaint, dysentery, and fever, appears to have been extensive in every part of the world, and he extolls nitric acid, and the new remedies, equally in these diseases as in venereal complaints. We suspect that a short residence in England will convince Mr. M'G. that his opinions on these remedies are now old fashioned.

Some cases of tetanus are adduced, which Mr. M'G. treated successfully with the warm bath and mercury, and the volume concludes with some remarks on the yellow fever, and a table of the points of resemblance between that disease and plague. On this part much is not said, the subject is barely broached; but it opens a mine of the most curious investigation, and we doubt not it will be pursued. Mr. M'Gregor was, perhaps, the first who had extensive experience of yellow fever and of the plague, the two most fatal diseases that afflict mankind; and he hints that he is in possession of ample materials to have enlarged this volume.

---

*Cases of Small Pox, subsequent to Vaccination, with Facts and Observations, read before the Medical Society, at Portsmouth, March 29,* 1804. *Addressed to the Directors of the Vaccine Institution. By* WILLIAM GOLDSON, *Member of the Royal College of Surgeons, in London.* Portsea, 1804, pp. 71.

THE importance of every document relating to Vaccine Inoculation, and the interest which the very title of the pamphlet before us cannot fail to excite, will justify us to our readers in giving its contents somewhat at large. This we shall do in the order in which we find them in this treatise, which, previous to publication, was read before the quarterly meeting of the Medical Society of Portmouth, Portsea, and Gosport, March 29, 1804.

The medical men of this town and neighbourhood, it seems, have had had some little imputation thrown on their professional zeal, from the tardiness with which they adopted vaccine inoculation, considering their proximity to the metropolis, and the ready communication with the head quarters of medical science which this part of the kingdom cannot fail to enjoy.

Vaccine Inoculation was even at last introduced in this place, according to Mr. Goldson's account, by *authority*, some matter being first sent by the Sick and Hurt Board (obtained we believe from the Vaccine Pock Institution) in the autumn of 1800, to Mr. Rickman, Surgeon of Marines, at Portsmouth, with instructions to try its efficacy on such recruits as had never had the small-pox. From this source Mr. G. first inoculated patients in November of the same year, and the practice soon spread pretty generally over the neighbourhood.

The date of the first inoculation at Portsmouth, (three years and a half from the present time) is surely a sufficient plea to exculpate the medical public of this part of the kingdom from any serious charge of indifference to professional improvement; nor is the plea much strengthened by such arguments as the following.

" The doctrine of the cow-pox was known to them soon after its promulgation. They attended to it with a desire to make themselves masters of the subject. At the same time, they could not remain ignorant of the many instances of failure, which occurred in its infancy. Neither could they help remarking, what must have been obvious to every attentive observer—the apparent instability of the practice. With every fresh instance of a spurious case they heard of new instructions and cautions in respect to taking the matter. These instructions deviated occasionally, from the 13th down to the 7th or 8th day; and yet they were told, that on this point depended the whole success of the operation. Besides, their local situation prevented them from having any opportunity to see the disease. Common prudence therefore, in a case so important, dictated, that they should not rashly venture on a practice, so seemingly replete with difficulties, the detection of which wholly depended upon experience alone. The vaccine pustule had not been seen by any of them, except in the representation of an engraving. Although those engravings were, most assuredly, very accurate, and the instructions equally explicit; yet, it must be acknowledged, there are many casual circumstances in pathology, which neither engravings nor instructions, however accurate, can convey a perfect idea of, and which can only be obtained by clinical attendance."

Spurious cases, fatal cases, and instances of failure, have been uniformly reported from the beginning of the practice to the present instant, and the opinions of experienced inoculators (as far as a very few years can give experience) have been constantly and are still at variance as to the time of taking, and mode of preserving matter.

Had these circumstances therefore been deemed of sufficient importance importance to delay the introduction of the new practice, it would still have been unknown and untried in this as in every other part of the country distant from the spot where the first experiments were made. The want of personal experience is a still more singular cause of hesitation, when the descriptions of the disease, both by writing and engraving, were confessedly accurate, and only deficient in "certain casual circumstances in pathology," which could not be known but by multiplied experiments, carried on by men of talents and observation in every variety of situation. Where was the personal experience of those medical men, (many of them in no respect inferior to the Portsmouth practitioners in sound judgement and prudent caution) who did not hesitate to begin vaccine inoculation with no other materials than the publications of Jenner, Woodville, and Pearson, and a few glasses of vaccine matter?

But to proceed with the author's cases.

The first case is the child of Mr. Grant, who was inoculated with cow-pox in October 1800, by Mr. Paytherus, and again inoculated in December 1803, by the author, with small-pox matter by way of proof of the efficacy of the first inoculation, which was acknowledged by Mr. Paytherus to be in every respect the regular vaccine disease. The variolous inoculation produced an inflamed pustule of an ambiguous appearance, which for six days was unattended with fever though the local inflammation was considerable, so that the author at first considered it as "a strong effort to produce small-pox," but no more. But on the eighth day the symptoms became more marked.

"Immediately on seeing the arm however on the next morning, Monday the 26th, I observed a visible alteration. The suppuration was manifestly increased. The areola was become extremely florid, and radiated, so as to be much less circumscribed, than it had hitherto been, bearing evident marks of absorption. The child was pale, not warmer than usual, but its pulse were quicker than they should have been, or than they ever had been before.

"These observations I kept to myself, as I perceived the anxiety of the parents led them to watch me with an inquisitive eye. But when I asked them, whether the child had been ill during the night, or whether they had observed any kind of appearance on the body, they instantly shewed me six or seven eruptions, Three of them were on the forehead and temple, one on the right ala of the nose, one on the opposite shoulder, and one or two on the breast. The child had been rather feverish during the early part of the night with restlessness, and according to the servants account transiently delirious.

"In the evening I was sent for in haste. I found it had been seized with a violent rigor, from which the attendants had, with difficulty, recovered him by warm wine and flannels. When I saw him, he was in a high degree of fever, his countenance much flushed, and there was a considerable efflorescence on both arms. It had the same characteristic appearance as the rash, which is

 frequently frequently seen in inoculated small-pox. Two or three eruptions, of the same kind as those seen in the morning, were readily distinguished through the efflorescence. The degree of fever was so much, that I thought it necessary to order some medicine to abate it, with a gentle anodyne to allay the irritation."

After this the fever abated, and in a few days the eruptions disappeared. The disease was considered by several medical gentlemen in the neighbourhood as well as by Mr. Goldson, as decidedly variolous.

This case gave rise to some correspondence with Mr. Paytherus, who had vaccinated the child, with Dr. Willan, and Mr. Ring, who agreed that the second disease was *not* the true small-pox, but an anomalous train of symptoms induced by irritation of the skin from the introduction of variolous matter, and which may happen at any time to persons who have already had the small-pox.

The second case is that of a child vaccinated in December 1800, and exposed to small-pox in February 1804, not by inoculation but by contagion, sleeping with another child under variolous inoculation. On a Thursday some time after this exposure the vaccinated child was attacked with fever, which continued till Sunday, when seven distinct eruptions appeared on the face, neck, and arms, which were pronounced by three respectable practitioners in Portsea to be decidedly small-pox. The eruptions continued five days but never maturated.

Mr. G. remarks, that here they were not attended with rash, and that in this respect the similarity to small-pox is as remarkable as in the former case, the natural disease being less frequently attended with efflorescence than the inoculated.

The third case is perhaps the most important. An infant was vaccinated in January 1801, went through the disease regularly, and matter taken from the same was employed with success to vaccinate another child, who has proved its efficacy by resisting small-pox repeatedly. In April 1803, the former child was exposed as much as possible to the contagion of small-pox, and compleatly resisted it. On March 1804, Mr. G. was desired to see the same child, who had then several eruptions on the body. Their history was the following.

"Not having been called early enough to witness the beginning and progress of the disease, I was the more particular in my inquiries. This I found was the fourth day of the eruption; she was taken ill on the Wednesday evening preceding, complaining of sickness, pain in the head and back, accompanied with considerable fever. On Thursday and part of Friday, she continued nearly the same. Supposing it to arise from cold, the mother was not alarmed, but gave her some diluting drink, and kept her in bed. About Friday noon she began to be better, but not totally free from fever. On Saturday morning she was perfectly recovered; but while she was dressing, a few eruptions were perceived in her face, neck, and shoulders, but were not much attended to at the time. On Sunday

2 the the number increased, and still more came out on Monday morning. They now began to consider them as something more than pimples. For the first time they suspected small-pox. In this they were justified, from variolous infection being in the school; two or three other children having taken it, one of which died, in a confluent sort, under my care soon after. This induced them to send for me.

"In a case so important, I should not have been justified in trusting to my own opinion. The case was therefore seen by Dr. Kerr of the Military Hospital, Dr. Thompson and Mr. Stevenson of Haslar, Mr. Rickman of the Marine Infirmary, Mr. Taswell and Mr. Merritt of Portsmouth; and by Mr. Gaselee, Mr. Hill, Mr. Seeds, and Mr. Weymouth of Portsea; all of whom expressed themselves perfectly satisfied of its being small-pox. Mr. Wilkinson of Portsmouth likewise saw the child, but entertained some doubts from the pustules drying off early on the seventh day. These doubts were removed however by the subsequent experiments."

The subsequent experiments were the following: Four children were inoculated by different practitioners with matter taken from the pustules of the abovementioned case, and with the following result.

"My own patient was a delicate child, about six months old. It had considerable fever and rash, which was preceded by two or three convulsions. The rash subsided about the usual time; when I could not discover more than eight or ten eruptions; four of which maturated, and went off on the seventh day.

"The child which Mr. Weymouth inoculated was ten months old. It had about fifty eruptions, most of which maturated kindly, and went off about the same time.

"The child upon whom Mr. Cooper tried the matter, was nearly of the same age. It was followed by more than a hundred pustules, which, like the former cases, went off on the seventh day.

"Mr. Seed's patient was a strong plethoric child at the breast, seven or eight months old. This child had considerable fever, with extensive rash, and more than a thousand pustules; most of which did not turn until the ninth or tenth day."

These cases being selected for the express purpose of experiment, were attended to by most if not all of the gentlemen who had visited the former case, and all were perfectly satisfied of their being variolous.

The above are the Author's cases; two others, attended with similar results, are given from the authority and personal observation of Mr. Weymouth of Portsea, in which, after satisfactory vaccination, a subsequent inoculation of small-pox produced fever, a regular pustule at the inoculated part, and in one of the cases a pustular eruption containing a virus, which by farther inoculation was found to produce a disease in every respect similar to genuine small-pox.

The author then enumerates the circumstances which have decided to his entire conviction the two great questions implicated in these cases, namely, whether the first inoculation produced the genuine and perfect vaccine disease, and whether the subsequent disease acquired from small-pox patients, either by inoculation or by contagion, was true and complete small-pox.

The original source of the vaccine matter sent to Portmouth appears unexceptionable. It was transmitted by the Sick and Hurt Board for the express purpose of experiment; the persons inoculated therewith, exhibited a well-defined pustule with all the characteristic marks of cow-pox, leaving a permanent scar, and rendering the persons thus affected, *for a time*, not susceptible of small-pox infection either by inoculation or contagion.

In answer to the question, whether the subsequent disease in the cases above mentioned was genuine small-pox, the author gives the following statement.

" Respecting the small-pox in Case II. Worsfold's child :—

" 1. Three years and three months after vaccination, she was exposed to infection from a child in the same house, who was inoculated for that disease.

" 2. There appeared seven or eight eruptions, attended with all the symptoms which usually precede, and accompany small-pox, exactly at the period when such infection might be expected to take place. They went off in a few days without maturating, as frequently happens in the inoculated disease.

" Respecting the small-pox in Case III. Luscombe's child :—

" 1. Three years after vaccination, she was exposed to variolous infection, in a school where several of the children were taken ill of that disorder, one of which died of it under my own care, and three others, as I have been informed, experienced the same fate.

" 2. There was an eruption of above a hundred pustules, attended with the usual symptoms of small-pox, some of which fully maturated, and continued to the seventh day, the others going off rather earlier without maturation, as frequently happens in distinct warty small-pox.

" 3. Matter taken from these pustules communicated the small-pox to four patients, under four different practitioners. In that under my own care, it produced but a few pustules, in two other cases, from fifty to a hundred, and in the fourth, above a thousand, accompanied with protracted disease. In all of them the pustules corresponded exactly with those of genuine small-pox. Therefore in both the subjects of Case II. and III. I consider the disease to be truly variolous."

The above is an abstract of the Author's evidence of the insufficiency of common vaccine inoculation; the entire Pamphlet claims an attentive perusal from all the partisans, friends, and well-wishers of Dr. Jenner's discovery.

Among the objections that have at various times been brought against vaccination, its insufficiency for permanent security has been sometimes urged, but no very precise attempt had been made, we believe, to give this opinion the support of actual experiment, before the cases related in the present publication.

The

The promoters of vaccination, after having quieted the scruples of the hesitating wavering parent on the supposed risk to life by cow-pox inoculation, the probability of introducing foreign disease, and the general security against future small pox, must often have heard the question, "How do I know that my child is secure for life? Will it not be necessary to re-inoculate him every three, four, or five years, to preserve in his constitution the same power of resisting small-pox infection?" Medical men have hitherto not scrupled to answer to such objections, that the strong analogy of cow-pox with the disease which it prevents, renders it highly probable that the security it affords from future infection, remains for life precisely as complete as immediately after inoculation: that, as it is the regular progress of small-pox inoculation, and not the quantity of disease, which ensures the future safety of the patient, so it is highly probable that the same law of the constitution obtains in vaccination; and especially, that positive experience proves that in casual vaccination, or wherever it is taken immediately from the cow, a lapse of many years, of half a century, in no degree diminishes its preservative power. Moreover, as it has never been pretended that the vaccine inoculation was in point of security from future small-pox, *preferable* to the variolous, it should be borne in mind, that in many constitutions the security produced even by previous small pox, however severe, is only comparative, and does not prevent the subsequent accession of fever, and the maturation of small-pox pustules after exposure to powerful infection.

The objections of Mr. Goldson, if valid, would go to the entire abolition of vaccine inoculation taken from the human subject; for the inference deduced from his statement would be, that the security given by such inoculation, though at first compleat, is every year gradually diminishing, not merely to a certain point of susceptibility to small-pox, but finally to the same degree of insecurity as if no inoculation had ever been performed.

The author, aware of the permanent value of vaccination *immemediately from the cow*, makes an exception to this species of cow-pox, and of course attaches the highest importance to the slight, but well defined, difference in colour and appearance which the two species exhibit. He likewise supposes that the usual place of casual vaccination, the hand, is particularly susceptible to irritating causes when the cuticle is abraded, and hence perhaps more efficacious. In this point however, we believe he is mistaken, as, if we recollect right, casual vaccination on the forehead, the shoulder, and other parts, has proved a permanent security for an indefinite number of years.

The author dedicates his treatise to the Vaccine (Vaccine-Pock) Institution, with peculiar propriety, as the original matter was furnished by this society, and as the duration of their practice affords them an opportunity of instituting further experiments on this infinitely important subject.

### *Dr. Trotter's Essay on Drunkenness,*

( Continued from Vol. xi. pp. 568—570.)

After instancing the effects of intoxication in rendering persons unsusceptible of cold, and insensible to pain, Dr. T. in a cursory way, adverts to the very different manner in which different nations are affected by ebriety. The next subject of investigation is the *chemical* effect of alkohol on the fluids and solids of the human body.

" Alkohol, certainly, deoxygenates the blood in some degree; at least, decompounds its floridity. The arterial blood of a professed drunkard approaches to the colour of venous; it is darker than usual. The rosy colour of the eruptions about the nose and cheeks does not disprove this; for it is probable that these spots attract oxygen from the atmosphere through the cuticle that covers them, just as Dr. Priestley observed venous blood, confined in a bladder, to acquire a more florid colour from the exposure to his dephlogisticated air. In the sea scurvy, a disease where, in the advanced stage, the blood is always found of a very dark colour, we know that spirituous liquors, more than any thing else, have a manifest tendency to aggravate every symptom. This fact has often come under my observation; and a very correct statement of the kind is to be found in my first volume on the Diseases of the Fleet, page 410.

" The component parts of alkohol are not sufficiently known; but it has a large proportion of hydrogen, which is proved by its combustion in pure air, when water is produced. Thus fourteen ounces of alkohol burnt in a proper apparatus, with a sufficient quantity of oxygen gas, yield sixteen ounces of pure water; hydrogen and oxygen being the component principles of water, as proved by modern chemistry. Alkohol has a strong attraction for water, and readily mixes with it, and it is the chief vehicle in which it is drank; but in what manner it is separated from the water within the body, would be difficult to find out. The evolution of hydrogenous gas is chiefly learned from the fœtor of the breath; it seems to be sent off from the surface of the lungs, in a disengaged state; and is often so pure in its kind from the expiration of a dram-drinker, that it is easily inflamed on the approach of a candle. The process of respiration probably effects this; and I should think at such a time there must be an unusual consumption of vital air. No experiments have been made on the blood of inebriates; and we are not informed, that in the circulating state, it exceeds the common temperature of the human body. But it is said, on the authority of Mr. Spalding, the celebrated diver, that after drinking spirits he always found the air in his bell consumed in a shorter time than when he drank water. This gentleman was lost in Dublin bay in 1783, in attempting to take the treasure out of an imperial Indiamen that sunk there, on her passage from Liverpool, where she was built: the misfortune, it appeared, was owing to the negligence of the attendants in not renewing the air.

" If

"If the blood of drunkards is strongly charged with hydrogen, must not that very much affect the quality of the biliary secretion, independent of any effect it may have on the liver itself? Might not the resinous matter which bile is found to contain, be greatly increased after spirituous potation? The liver is an organ very liable to be injured by hard drinking; this gives cause for suspicion, that the *chemical* operation of alkohol on the blood and the bile, has also some share in producing hepatic diseases. It may increase the generation of *biliary calculi*, and the disposition to dyspepsia, which prevail in the constitution of drunkards.

"Is the perspirable matter of drunkards at all impregnated with hydrogenous gas?

"I am much of opinion that the *chemical* operation of alkohol, has a great influence in retarding the healing of wounds, and in converting them into ulcers. I believe all surgeons agree, that such an effect takes place after hard drinking, though it is generally attributed to the fever and inflammation which it occasions. The common appearance of eruptions on the surface of the body, may in a great measure be referred to the same source. The exhalations of hydrogenous gas, which arise in some places, are very apt to irritate the eyes, and bring on a painful ophthalmia; from which it is fair to infer, that the same effect may take place, from blood loaded with hydrogen, circulating through the minute vessels of the *tunica adnata*, as the disease is a common one with wine-bibbers. The fœtor of ulcers, in all drunken subjects, is unusually great; and I shall speak of this under the diseases.

"But the most interesting part of this doctrine, is the *combustion of the human body*, produced by the long and immoderate use of spirituous liquors. Such cases are on record; and a collection of them, with remarks, is to be found in the *Journal de Physic*, year 8, by Pierre Aime Lair. I subjoin a copy of that memoir, taken from the Philosophical Magazine, vol. vi. p. 132, by Mr. Alexander Tilloch."

Then follows a considerable number of instances of persons who had been addicted to the immoderate use of spirits, undergoing such combustion; and Dr. T. concludes that part of his subject with the following reflections.

"I shall not extend further these observations on the combustion of the human body, as I flatter myself that after this examination every person must be struck with the relation which exists between the cause of this phænomenon and the effects that ensue. A sytem embellished with imaginary charms is often seducing, but it never presents a perfect whole. We have seen facts justify reasoning, and reasoning serve afterwards to explain facts. The combustion of the human body, which, on the first view, appears to have in it something of the marvellous, when explained exhibits nothing but the utmost simplicity: so true it is, that the wonderful is often produced by effects which, as they rarely strike our eyes, permit our minds so much the less to discover their real cause.

"Some people may, however, ascribe to the wickedness of mankind what we ascribe to accident. It may be said that assassins, after putiing to death their unfortunate victims, rubbed over their bodies with combustible substances, by which they were consumed. But even if such an idea should ever be conceived, it would be impossible to carry it into execution. Formerly, when criminals were condemned to the flames, what a quantity of combustible substances was necessary to burn their bodies? A baker's boy, named Renaud, being condemned to be burnt a few years ago at Caen, two large cart-loads of faggots were required to consume the body, and at the end of more than ten hours some remains of the bones were still to be seen. What proves that the combustion in the before-mentioned instances was not artificial is, that people often arrived at the moment when it had taken place, and that the body was found in its natural state. People entered the house of Madame Boiseon at the time when her body was on fire, and all the neighbours saw it. Besides, the people of whom I have spoken were almost all of the lowest class, and not much calculated to give rise to the commission of such a crime. The woman mentioned in the transactions of Copenhagen was of the poorest condition; Grace Pitt was the wife of a fishmonger; Mary Jauffret, that of a shoemaker; and two other women, who resided at Caen, belonged to the lowest order of society. It is incontestible, then, that in the instancess I have adduced, the combustion was always accidental, and never intentional.

"It may be seen, that a knowledge of the causes of this phænomenon is no less interesting to criminal justice than to natural history, for unjust suspicions may sometimes fall on an innocent man. Who will not shudder on recollecting the unfortunate inhabitant of Rheims, who after having lost his wife by the effect of combustion, was in danger of perishing himself on the scaffold, condemned unjustly by an ignorant tribunal!

"I shall consider myself happy if this picture of the fatal effects of intoxication makes an impression on those addicted to this vice, and particularly on women, who most frequently become the victims of it. Perhaps the frightful details of so horrid an evil as that of combustion will reclaim drunkards from this horrid practice. Plutarch relates, that at Sparta, children were deterred from drunkenness by exhibiting to them the spectacle of intoxicated slaves, who, by their hideous contortions, filled the minds of these young spectators with so much contempt that they never afterwards got drunk. This state of drunkenness, however, was only transitory. How much more horrid it appears in those unfortunate victims consumed by the flames and reduced to ashes! May men never forget that the vine sometimes produces very bitter fruit,—disease, pain, repentance, and DEATH!

How

*How far are the Acts of the Drunkard to be palliated?*

"This is a point of great importance in civilized society: but it is not the province of the physician to decide with a legal view. Every human being, who was ever intoxicated, must have found, on reflection, that he had said and done things which he would have neither thought of nor acted in a state of sobriety. The peace of his neighbour has, therefore, required that the drunkard should answer for his conduct. But it may be asked, ought a madman to answer for his deeds? Certainly. The man who becomes mad from immoderate vinous potation must be amenable to law, because that madness was of his own seeking. Again, it may be said, that the drunken man, being as much in a state of delirium as any maniac, ought he to be punished for doing what he is unconscious of? Yes: But punishment might be mitigated here, if it shall appear that no preconceived malice had prompted him. This is, I think, what lawyers call *mal propense*. Were a man, during ebriety, to sign a deed, by which he should dispose of his property in an improper manner, to the injury of his family; quere, would such a deed be legal? It might be deemed legal; but to me it would appear unjust to confirm it; because the man never formed such a resolution when he was in his senses. The acts of the drunkard, in this respect, ought not to be valid: for this plain reason, in the same condition he is not allowed to injure his neighbour, or society at large, with impunity; and therefore he ought not to be permitted to injure either his family or himself. All debts incurred, or money lost at play, in the state of intoxication, ought to be declared *null*, on the loser appealing in a proper manner when sober. This would prevent the gamester and systematic villain from taking advantage of the honest man, and would correct some of the greatest evils in the community.

"When a drunken man is lavish of promises which he never made when sober; be assured his kindness is not worth your thanks.

"When you hear a drunken man boasting of his generosity to his friends; beware, how you a receive a favour from that man.

"When you hear a drunken man telling family secrets, whether of his own, or those of other people; put that man down for a fool; and take care what you say in his presence.

"When you hear a drunken man boasting of his favours from the *sex;* be assured, that man has no honour.

"When you hear a drunken man bragging of his courage; mark that man a coward

"When you hear a drunken man vaunting of his riches; be assured, he cannot be estimable for his virtues.

"When you hear a drunken man pitying misfortunes which he did not relieve when sober; it is the strongest proof that he possesses no goodness of heart.

"Receive no donations from a drunken man; lest he should ask them again when sober.

"Avoid the company of a drunkard; for if he insults you, and you you should insist on satisfaction, he will plead want of recollection, as apology.

"Let the sober man beware of the society of drunkards, lest the world should say, that he means to take an advantage of their credulity."

